# Associations of serum long-chain fatty acids with multiple organ involvement in patients with sarcoidosis

**DOI:** 10.1186/s12890-022-02084-x

**Published:** 2022-07-28

**Authors:** Takahito Suzuki, Masato Karayama, Yusuke Inoue, Hironao Hozumi, Yuzo Suzuki, Kazuki Furuhashi, Tomoyuki Fujisawa, Noriyuki Enomoto, Yutaro Nakamura, Naoki Inui, Takafumi Suda

**Affiliations:** 1 Second Division, Department of Internal Medicine, Hamamatsu University School of Medicine, 1-20-1 Handayama, Hamamatsu, 431-3192 Japan; 2 Department of Chemotherapy, Hamamatsu University School of Medicine, 1-20-1 Handayama, Hamamatsu, 431-3192 Japan; 3 Department of Clinical Pharmacology and Therapeutics, Hamamatsu University School of Medicine, 1-20-1 Handayama, Hamamatsu, 431-3192 Japan

**Keywords:** Lipid, Metabolites, omega-3 fatty acids, omega-6 fatty acids, Immune metabolism

## Abstract

**Background:**

Fatty acids have diverse immunomodulatory functions and the potential to be associated with inflammatory responses in sarcoidosis.

**Methods:**

The serum levels of multiple long-chain fatty acids (LCFAs) were compared between 63 patients with sarcoidosis and 38 healthy controls. The associations of LCFAs with clinical outcomes of sarcoidosis were also evaluated.

**Results:**

The patients with sarcoidosis had significantly lower levels of n-3 poly-unsaturated fatty acids (PUFAs) (*p* < 0.001) and n-6 PUFAs (*p* < 0.001) than the healthy controls. However, there were no significant differences in the levels of saturated fatty acids (SFAs) and mono-unsaturated fatty acids (MUFAs) between the two groups. On multivariate logistic analysis, lower levels of n-3 PUFAs, n-6 PUFAs, and n-3/n-6 ratio were predictive of sarcoidosis. Among the patients with sarcoidosis, those with multiple organ involvement had significantly lower levels of n-3 PUFAs and n-3/n-6 ratio than those with single organ involvement. There were no significant differences in the levels of n-6 PUFAs, SFAs, and MUFAs between the patients with multiple and single organ involvement. On multivariate logistic analysis, lower levels of SFAs and n-3/n-6 ratio were predictive of multiple organ involvement. The levels of LCFAs had no significant association with radiographic stage or spontaneous remission.

**Conclusions:**

Assessment of LCFA profiles may be useful for the diagnosis of sarcoidosis and evaluation of the disease activity.

**Supplementary Information:**

The online version contains supplementary material available at 10.1186/s12890-022-02084-x.

## Introduction

Sarcoidosis is a systemic granulomatous disease that affects multiple organs and lymphatic systems throughout the body [1]. It is widely considered to be a benign disease, and spontaneous remission occurs in about two-thirds of patients. However, patients with progressive pulmonary disease, central nerve, or heart involvement have a poor prognosis [1, 2]. Although the precise mechanism remains unknown, sarcoidosis is thought to result from a systemic inflammatory disorder involving Th1 immune responses [1, 3]. Recent evidence supports the notion that sarcoidosis involves complex interplay among diverse immune cells [3].

It has become increasingly clear that metabolites derived from nutrients like glucose, amino acids, and fatty acids play essential roles in the control of immunity. Fatty acids not only serve as energy resources and cell membrane components, but also act for activation of immune cells, including effector T cells, natural killer T cells, macrophages, and dendritic cells [4, 5, 6, 7, 8, 9]. Fatty acids are divided into several groups based on common molecular structures and specific immunomodulatory properties. For example, lipid mediators derived from n-6 polyunsaturated fatty acids (PUFAs), such as prostaglandin D_2_ (PGD_2_), PGE_2_, and leukotriene B4 (LTB_4_), have pro-inflammatory properties, while lipid mediators derived from n-3 PUFAs, such as maresins and resolvins, have anti-inflammatory properties [10, 11]. Furthermore, saturated fatty acids (SFAs) can induce polarization of naïve T cells toward Th1 and Th17 cells [12, 13], activate bone marrow-derived dendritic cells, and increase T cell activation capacity [14].

Because of their immunomodulatory roles, fatty acids have attracted attention as the pathogenesis and/or potential treatment targets in several inflammatory diseases. For example, patients with chronic obstructive pulmonary disease (COPD) had lower levels of n-3 PUFAs in their sputum than healthy controls [15]. In addition, higher levels of n-6 PUFAs were observed during acute exacerbation of COPD compared with stable COPD [15]. Furthermore, patients with idiopathic pulmonary fibrosis had higher levels of SFAs in their bronchoalveolar lavage fluid than healthy controls [16]. Meanwhile, epidemiological data indicated a beneficial effect of n-3 PUFAs for prevention of ulcerative colitis, while consumption of a lower ratio of n-3 PUFAs to n-6 PUFAs was associated with an increased risk of ulcerative colitis [17].

Nevertheless, little is known about the fatty acid profiles in patients with sarcoidosis. Given the diverse immunomodulatory functions of fatty acids and their associations with several inflammatory diseases, we hypothesized that altered profiles of fatty acids are associated with sarcoidosis and disease severity. In the present study, we compared the serum levels of multiple fatty acids between patients with sarcoidosis and healthy controls. We also evaluated the associations of fatty acid levels with disease severity and clinical outcomes of sarcoidosis.

## Methods

### Participants

This retrospective observational study was conducted in accordance with the ethical standards of the Declaration of Helsinki. Consecutive patients with sarcoidosis diagnosed between May 1998 and December 2019 at Hamamatsu University Hospital were included. The diagnosis of sarcoidosis was based on the consensus statement of the American Thoracic Society, the European Respiratory Society, and the World Association of Sarcoidosis and Other Granulomatous Disorders [1]. Eligible patients were required to have available fasting serum samples at diagnosis. The requirement for informed consent from the patients was waived because of the retrospective nature of the study. Healthy volunteers without sarcoidosis were evaluated as controls. The study was approved by the Institutional Review Board of Hamamatsu University School of Medicine (No. 20–228).

### Data collection

Clinical data at diagnosis, including age, sex, body mass index (BMI), smoking status, affected organs, laboratory parameters, radiographic data, pulmonary function tests, bronchoalveolar lavage (BAL), pathological information, and affected organs, were retrospectively evaluated using medical records. Radiographic stage was evaluated according to the consensus statement of the American Thoracic Society, the European Respiratory Society and the World Association of Sarcoidosis and Other Granulomatous Diseases [1]. Treatments and clinical courses were also evaluated during the study period. For the healthy controls, age, sex, BMI, and smoking status were evaluated. Disease activity was defined as follows: 1) progressive disease, (a) decrease in forced vital capacity (FVC) and forced expiratory volume in 1 s (FEV_1_) of > 10% from baseline, (b) decrease in FVC or FEV_1_ of > 10% from baseline with worsening of pulmonary symptoms, (c) worsening of radiologic findings in pulmonary or extrapulmonary lesions, or (d) development of new organ involvement; 2) improved disease, (a) increase in FVC and FEV_1_ of > 10% from baseline without worsening of pulmonary symptoms, (b) increase in FVC or FEV_1_ of > 10% from baseline with improvement of pulmonary symptoms, or (c) improvement of radiographic findings in pulmonary or extrapulmonary lesions: and 3) stable disease, none of the above. [18, 19, 20].

### LCFA measurements

Multiple LCFAs were evaluated using serum samples at the time of diagnosis. The LCFAs were evaluated with a gas chromatography system (GC-2010; Shimadzu, Kyoto, Japan), using a TC-70 column (GL Science, Tokyo, Japan; length: 30 m; caliber: 0.25 mm; film thickness: 0.25 μm), a carrier gas of helium, and a temperature elevation program from 80.0 °C to 200.0℃. Twenty-four LCFAs were evaluated as follows: (1) n-3 PUFAs = linolenic acid, eicosapentaenoic acid, docosapentaenoic acid, docosahexaenoic acid; (2) n-6 PUFAs = linoleic acid, γ-linolenic acid, eicosadienoic acid, dihomo-γ-linolenic acid, arachidonic acid, docosatetraenoic acid; (3) SFAs = lauric acid, myristic acid, palmitic acid, stearic acid, arachidic acid, behenic acid, lignoceric acid; and (4) monounsaturated fatty acids (MUFAs) = myristoleic acid, palmitoleic acid, oleic acid, eicosenoic acid, eicosatrienoic acid, erucic acid, nervonic acid. Erucic acid was excluded from the analysis because its levels were below the measurement sensitivity in most patients. The levels of the LCFAs were evaluated separately and together in each category. The ratio of n-3 and n-6 PUFAs (n-3/n-6 ratio) was also calculated to evaluate the balance of these PUFAs. All LCFAs were measured at a laboratory (SRL Inc., Tokyo, Japan) certified by the College of American Pathologists and International Organization for Standardization 15,189.

### Statistical analyses

The Mann–Whitney U-test and Fisher’s exact test were used for comparisons of continuous and categorical variables, respectively. Correlations between LCFA levels and clinical data were assessed using Spearman’s rank correlation coefficient. Receiver operating characteristic curve analyses were conducted to determine cutoff values for LCFA levels using the Youden Index (maximum value of [sensitivity + specificity − 1]) (Additional file1: Tables S1 and S2). Logistic regression analyses were carried out to evaluate predictive factors for the diagnosis of sarcoidosis and multiple organ involvement. Age, sex, and variables with *P* < 0.100 in univariate analyses were employed for multivariate analyses. When variables had strong correlations with one another (Spearman’s correlation coefficient > 0.7), only one was selected for multivariate analysis to avoid multicollinearity. Candidate combinations for multivariate analysis were created using LCFAs without strong correlations with one another. Akaike information criteria were used to obtain estimated prediction errors for the multivariate analyses. Statistical analyses were performed using EZR software (version 1.41; Saitama Medical Center, Jichi Medical University, Saitama, Japan). Values of *P* < 0.05 were considered statistically significant.

## Results

### Patient characteristics

**Fig. 1:**
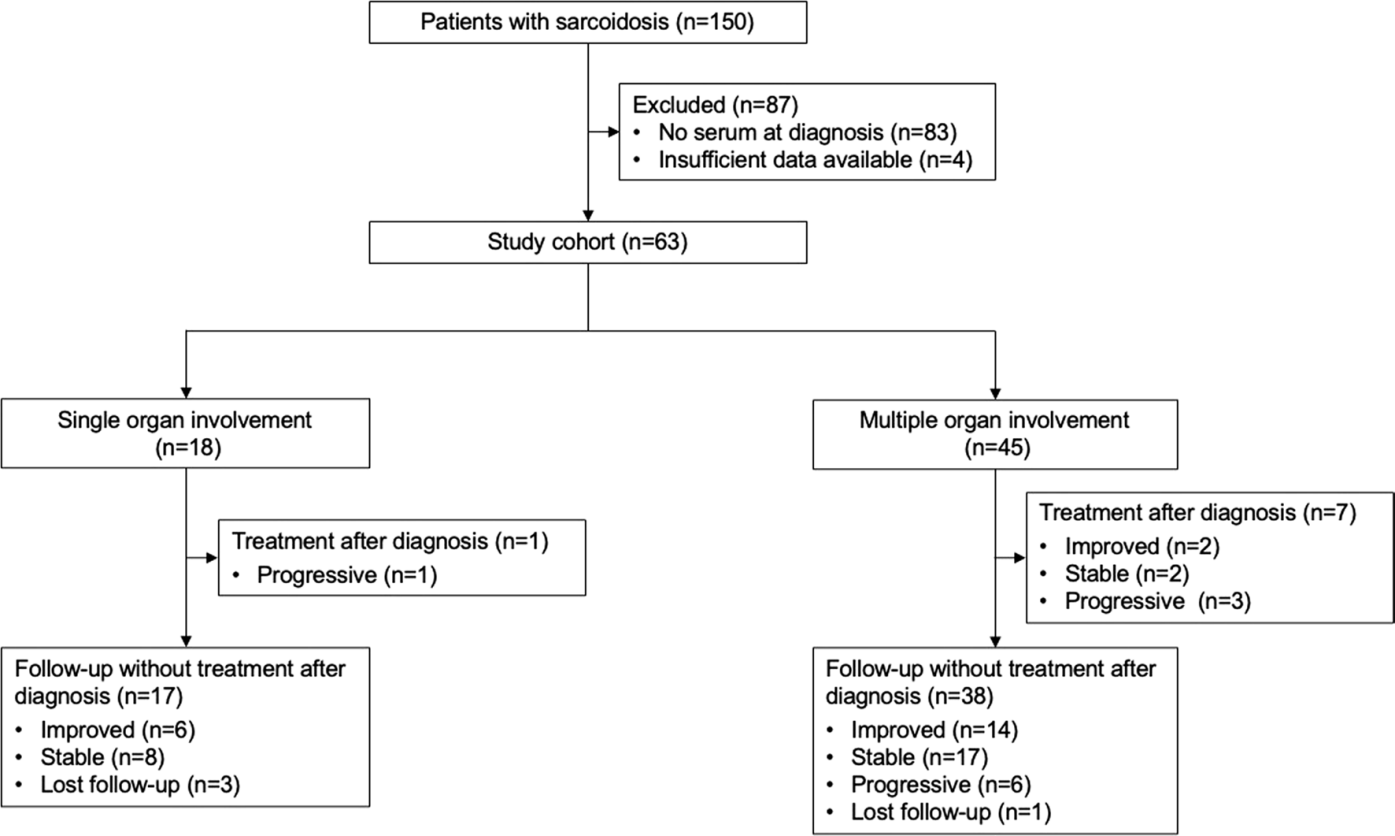
Flow chart diagram for the study

A total of 150 patients with sarcoidosis were screened, of whom 87 were excluded because they lacked assessable serum samples at diagnosis (*n* = 83) or had insufficient available data (*n* = 4). As a result, 63 patients with sarcoidosis were included in the study (Fig. 1). The patient characteristics are shown in Table 1. The median age was 58 years (interquartile range [IQR], 45–68 years) and 38 (60.3%) patients were female. Fifty-four (85.7%) patients had a histological diagnosis of sarcoidosis. The most frequently affected organs were the lungs (98.4%), followed by the eyes (52.4%), skin (15.9%), and heart (6.3%). Forty-five (71.4%) patients had two or more affected organs. On chest X-rays, 21 (33.3%), 40 (63.5%), and 36 (57.1%) patients had bilateral hilar lymphadenopathy (BHL), lung parenchymal involvement, or both, respectively. Among 61 patients who underwent pulmonary function tests, 55 (90.2%) had normal pulmonary function (forced vital capacity, % predicted [%FVC] ≥ 80.0). The median observation time was 5.5 years (IQR, 4.0–10.3 years). The 38 healthy controls had a median age of 34 years (IQR, 27–43 years), a median BMI of 22.2 kg/m^2^ (IQR, 19.6–23.7 kg/m^2^), a female proportion of 39.5%, and no smoking history (Additional file 1: Table S3). The healthy controls were significantly younger and had a lower proportion of smoking history than the patients with sarcoidosis (both *p* < 0.001).

**Table 1:** Patient characteristics

	All patients n = 63	Single organ involvement, n = 18	Multiple organ involvement n = 45	*P* value*
Age, years	58 (45–68)	59 (44–75)	57 (45–65)	0.330
Sex, female	38 (60.3)	9 (50.0)	29 (64.4)	0.394
Body mass index, kg/m^2^	21.4 (20.1–23.8)	21.9 (20.4–23.1)	21.4 (19.9–23.8)	0.897
Smoking, ever-smoker	31 (49.2)	9 (50.0)	22 (48.9)	1.000
Number of affected organs, 1/2/3/4/5	18/30/13/1/1	18/0/0/0/0	0/30/13/1/1	< 0.001
Affected organs				
Lungs^a^	62 (98.4)	17 (94.4)	45 (100.0)	0.286
Eyes	33 (52.4)	1 (5.6)	32 (71.1)	< 0.001
Skin	10 (15.9)	0	10 (22.2)	0.051
Heart	4 (6.3)	0	4 (8.9)	0.317
Others^b^	13 (20.6)	0	13 (28.9)	0.013
Histological diagnosis	54 (85.7)	16 (88.9)	38 (84.4)	1.000
Serum ACE, IU/L	19.3 (14.5–25.3)	16.6 (14.2–21.5)	21.4 (15.1–25.4)	0.122
Radiographic stage, 0/I/II/III/IV	1/21/36/4/1	1/6/9/1/1	0/15/27/3/0	0.802
Pulmonary function tests (n = 61)				
FVC, L	2.76 (2.34–3.40)	3.00 (2.43–3.49)	2.75 (2.33–3.30)	0.389
FVC, % predicted	94.9 (88.1-100.7)	96.4 (90.8-104.9)	94.1 (87.5-100.3)	0.187
FEV_1_, L	2.10 (1.82–2.79)	2.55 (1.92–3.36)	2.10 (1.81–2.66)	0.385
FEV_1_, % predicted	90.3 (77.4-100.2)	89.5 (78.2–98.8)	91.1 (76.1-100.5)	0.915
FEV_1_/FVC, %	77.9 (74.3–83.8)	78.2 (74.8–82.8)	77.9 (72.9–85.1)	0.812
Bronchoalveolar lavage (n = 62)				
Total cells, ×10^5^/ml	0.96 (0.61–1.48)	1.00 (0.51–1.51)	0.93 (0.68–1.47)	0.561
Lymphocytes, %	11.9 (8.2–22.9)	19.0 (5.9–26.5)	11.5 (8.3–19.9)	0.852
CD4/CD8 ratio	5.02 (3.30–7.75)	4.58 (3.30–6.49)	5.16 (3.34–7.91)	0.841
Treatment, none/CS/CS + azathioprine	50/12/1	17/1/0	33/11/1	0.190

Data are presented as median (interquartile range) or number (%)

ACE, angiotensin-converting enzyme; CS, corticosteroids; FVC, forced vital capacity; FEV_1_, forced expiratory volume in 1 s

Radiographic stages 0, I, II, III, and IV represent normal appearance, bilateral hilar lymphadenopathy (BHL) alone, BHL and lung parenchymal involvement, lung parenchymal involvement without BHL, and pulmonary fibrosis, respectively

^a^Lungs include pulmonary hilar and mediastinal lymphadenopathy

^b^Others include muscle, liver, thyroid gland, spleen, nerve, and extramediastinal lymphadenopathy

*Comparison between patients with single and multiple organ involvement

### Clinical outcomes of patients with sarcoidosis

Eight (12.7%) patients received treatment for sarcoidosis from the time of diagnosis, comprising 7 with corticosteroid alone and 1 with a combination of corticosteroid plus azathioprine. After the treatment, 2 had remission, 2 had stable disease, and 4 had disease progression (Fig. 1). The remaining 55 patients were initially followed without treatment, of whom 20 (36.4%) had spontaneous remission, 25 (45.5%) had stable disease, 6 (10.9%) had disease progression, and 4 (7.3%) were lost to follow-up during the observation period (Fig. 1). Among 6 patients with disease progression after the initial follow-up, 2 (33.3%), 3 (50.0%), and 1 (16.7%) had worsening of lung involvement, development of non-pulmonary lesions, and both, respectively, with 5 receiving corticosteroid alone and 1 receiving no treatment. Among the 5 patients who received corticosteroid, 4 had remission, but 1 had disease progression. One patient who did not receive treatment after disease progression did not deteriorate further or require any treatment.

### Associations of LCFA levels with demographic characteristics

Among the 63 patients with sarcoidosis, there were weak or moderate correlations between age and n-3 PUFAs (*r* = 0.35) or n-3/n-6 ratio (*r* = 0.49), and between BMI and MUFAs (*r* = 0.25) (Additional file 1: Fig. S1). The LCFA levels were not correlated with sex (Additional file 1: Table S4). Among the LCFAs, there were strong correlations between n-3 PUFAs and n-6 PUFAs (*r* = 0.80), and between SFAs and MUFAs (*r* = 0.92). The correlations among single LCFAs are presented in Additional file 1: Fig. S2.

### Comparisons of serum LCFA levels between patients with sarcoidosis and healthy controls

**Fig. 2:**
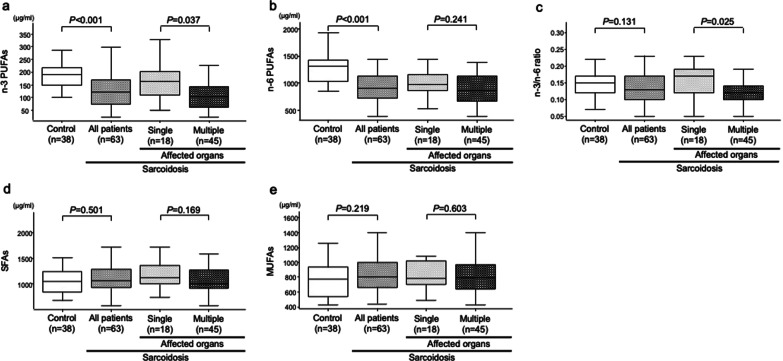
Levels of long-chain fatty acids in the patients with sarcoidosis and healthy controls. **A**–**E** Levels of n-3 poly-unsaturated fatty acids (PUFAs) (**A**), n-6 PUFAs (**B**), n-3/n-6 ratio (**C**), saturated fatty acids (SFAs) (**D**), and mono-unsaturated fatty acids (MUFAs) (**E**). The levels of serum fatty acids were measured by gas chromatography. The Mann–Whitney U-test was used to compare the levels of fatty acids. Gray, light gray, dark gray, and white data indicate all patients with sarcoidosis, patients with sarcoidosis and single organ involvement, patients with sarcoidosis and multiple organ involvement, and healthy controls, respectively. Horizontal lines, boxes, and error bars, represent the median, the 25th and 75th percentiles, and the 10th and 90th percentiles, respectively

The patients with sarcoidosis had significantly lower levels of n-3 PUFAs (*p* < 0.001) and n-6 PUFAs (*p* < 0.001) than the healthy controls (Fig. 2 A, B). When the LCFAs were evaluated separately, the levels of all n-3 PUFAs (linolenic acid, eicosapentaenoic acid, docosapentaenoic acid, docosahexaenoic acid) were significantly lower in the patients with sarcoidosis (*p* < 0.001 for all) (Additional file 1: Fig. S3). Likewise, the levels of all n-6 PUFAs other than eicosadienoic acid (linoleic acid, γ-linolenic acid, dihomo-γ-linolenic acid, arachidonic acid, docosatetraenoic acid) were significantly lower in the patients with sarcoidosis (*p* < 0.001, *p* = 0.016, *p* = 0.007, *p* < 0.001, and *p* < 0.001, respectively) (Additional file 1: Fig. S3). However, there were no significant differences in the levels of n-3/n-6 ratio, SFAs, and MUFAs between the two groups (Fig. 2 C–E). Furthermore, the levels of myristic acid (*p* = 0.004), myristoleic acid (*p* < 0.001), and palmitoleic acid (*p* < 0.001) were significantly higher in the patients with sarcoidosis, while the levels of other SFAs and MUFAs did not differ between the two groups (Additional file 1: Fig. S3).

On univariate logistic analysis, higher levels of MUFAs and lower levels of n-3 PUFAs, n-6 PUFAs, and n-3/n-6 ratio were predictive of sarcoidosis, as were age and sex (Table 2). On multivariate logistic analysis, lower levels of n-3 PUFAs, n-6 PUFAs, and n-3/n-6 ratio were predictive of sarcoidosis, as was age (Table 2). When the LCFAs were evaluated separately, higher levels of myristic acid, myristoleic acid, palmitoleic acid, and eicosenoic acid, and lower levels of arachidic acid, behenic acid, linolenic acid, eicosapentaenoic acid, docosapentaenoic acid, docosahexaenoic acid, linoleic acid, γ-linolenic acid, dihomo-γ-linolenic acid, arachidonic acid, and docosatetraenoic acid were predictive of sarcoidosis on univariate logistic analysis (Additional file 1: Table S5). After adjustment by age and sex, higher levels of myristoleic acid and palmitoleic acid, and lower levels of stearic acid, arachidic acid, behenic acid, lignoceric acid, linolenic acid, eicosapentaenoic acid, docosapentaenoic acid, docosahexaenoic acid, linoleic acid, γ-linolenic acid, dihomo-γ-linolenic acid, arachidonic acid, and docosatetraenoic acid were independently predictive of sarcoidosis (Additional file 1: Table S5).

**Table 2:** Logistic regression analyses for the diagnosis of sarcoidosis

	Univariate analysis		Multivariate analysis					
			Set A		Set B		Set C	
Variables	OR (95%CI)	*P*	OR (95%CI)	*P*	OR (95%CI)	*P*	OR (95%CI)	*P*
Age^a^	1.11 (1.06–1.16)	< 0.001	1.20 (1.10–1.31)	< 0.001	1.11 (1.05–1.18)	< 0.001	1.11 (1.06–1.16)	< 0.001
Sex, male	0.43 (0.19–0.98)	0.044	2.66 (0.44–16.30)	0.289	1.64 (0.38–7.01)	0.505	0.81 (0.26–2.59)	0.727
BMI^b^	0.97 (0.83–1.13)	0.671						
n-3 PUFAs, high	0.05 (0.01–0.18)	< 0.001	0.01 (0.00-0.05)	< 0.001				
n-6 PUFAs, high	0.06 (0.02–0.19)	< 0.001			0.03 (0.01–0.14)	< 0.001		
n-3/n-6 ratio, high	0.34 (0.13–0.90)	0.030					0.25 (0.07–0.83)	0.023
SFAs, high	2.18 (0.85–5.61)	0.105						
MUFAs, high	3.26 (1.14–9.34)	0.028	1.13 (0.15–8.71)	0.906	4.27 (0.87-21.00)	0.074	1.59 (0.38–6.58)	0.526

The cutoff value for each long-chain fatty acid was determined by the Youden Index in receiver operating characteristic curve analysis (Supplementary Table 1). The Akaike Information criteria for Sets A, B, and C were 58.6, 76.1, and 96.4, respectively. OR, odds ratio; CI, confidence interval; BMI, body mass index; SFAs, saturated fatty acids; PUFAs, polyunsaturated fatty acids; MUFAs, monounsaturated fatty acids

^a^Per 1-year increase

^b^Per 1-kg/m^2^ increase

## Associations of serum LCFA levels with multiple organ involvement in patients with sarcoidosis

Among the patients with sarcoidosis, those with multiple organ involvement had significantly lower levels of n-3 PUFAs and n-3/n-6 ratio than those with single organ involvement (Fig. 2 A, C). When the LCFAs were evaluated separately, the levels of all n-3 PUFAs except linolenic acid (eicosapentaenoic acid, docosapentaenoic acid, docosahexaenoic acid) were significantly lower in the patients with multiple organ involvement (*p* = 0.032, *p* = 0.021, and *p* = 0.033, respectively) (Additional file 1: Fig. S3). Meanwhile, there were no significant differences in the levels of n-6 PUFAs, SFAs, and MUFAs between the patients with multiple and single organ involvement (Fig. 2B, D, E).

On univariate logistic analysis, lower levels of SFAs, n-3 PUFAs, n-6 PUFAs, and n-3/n-6 ratio were predictive of multiple organ involvement (Table 3). On multivariate logistic analysis, lower levels of SFAs and n-3/n-6 ratio were predictive of multiple organ involvement (Table 3). When the LCFAs were evaluated separately, lower levels of palmitic acid, stearic acid, palmitoleic acid, eicosatrienoic acid, eicosapentaenoic acid, docosapentaenoic acid, docosahexaenoic acid, linoleic acid, γ-linolenic acid, and dihomo-γ-linolenic acid were predictive of multiple organ involvement on univariate logistic analysis, (Additional file 1: Table s6). After adjustment by age, sex, and serum angiotensin converting enzyme level, lower levels of palmitoleic acid, eicosatrienoic acid, eicosapentaenoic acid, docosapentaenoic acid, docosahexaenoic acid, γ-linolenic acid, and dihomo-γ-linolenic acid were independently predictive of multiple organ involvement (Additional file 1: Table S6).

**Table 3:** Logistic regression analyses for multiple organ involvement in sarcoidosis

	Univariate analysis		Multivariate analysis					
			Set 1		Set 2		Set 3	
Variables	OR (95% CI)	*P* value	OR (95% CI)	*P* value	OR (95% CI)	*P* value	OR (95% CI)	*P* value
Age^a^	0.98 (0.95–1.02)	0.339	0.99 (0.94–1.04)	0.716	0.98 (0.93–1.02)	0.300	1.01 (0.95–1.06)	0.815
Sex, male	0.55 (0.18–1.67)	0.293	0.64 (0.15–2.67)	0.537	0.53 (0.13–2.19)	0.383	0.72 (0.16–3.25)	0.664
BMI^b^	1.01 (0.82–1.25)	0.930						
Serum ACE, ≥ 21.4 IU/L	1.08 (0.99–1.18)	0.096	2.41 (0.64–9.14))	0.196	2.40 (0.64–9.03)	0.195	2.53 (0.62–10.30)	0.195
Radiographic stage, ≥ II	1.27 (0.41–3.95)	0.676						
Pulmonary function test								
FVC % predicted^c^	0.97 (0.93–1.02)	0.240						
FEV1/FVC^c^	1.00 (0.98–1.01)	0.692						
Bronchoalveolar lavage								
Lymphocytes^c^	1.01 (0.97–1.04)	0.767						
CD4/CD8 ratio, ≥ 3.5	0.99 (0.29–3.39)	0.992						
n-3 PUFAs, high	0.23 (0.07–0.74)	0.013	0.33 (0.09–1.24)	0.101				
n-6 PUFAs, high	0.30 (0.09–1.05)	0.059			0.43 (0.11–1.67)	0.225		
n-3/n-6 ratio, high	0.16 (0.05–0.53)	0.003					0.14 (0.03–0.61)	0.009
SFAs, high	0.16 (0.03–0.76)	0.022	0.25 (0.05–1.35)	0.107	0.25 (0.05–1.34)	0.105	0.16 (0.03–0.96)	0.045
MUFAs, high	1.00 (0.18–5.69)	1.000						

The cutoff value for each long-chain fatty acid was determined by the Youden Index in receiver operating characteristic curve analysis (Supplementary Table 2). The Akaike Information criteria for Sets 1, 2, and 3 were 74.1, 75.4, and 69.1, respectively. OR, odds ratio; CI, confidence interval; BMI, body mass index; ACE, angiotensin-converting enzyme; FVC, forced vital capacity; FEV_1_, forced expiratory volume in 1 s; SFAs, saturated fatty acids; PUFAs, polyunsaturated fatty acids; MUFAs, monounsaturated fatty acids

^a^Per 1-year increase

^b^Per 1-kg/m^2^ increase

^c^Per 1% increase

## Associations of serum LCFA levels with other outcomes in patients with sarcoidosis

There were no significant associations between lung parenchymal involvement (radiographic stage ≥ 2) and n-3 PUFAs, n-6 PUFAs, n-3/n-6 ratio, SFAs, or MUFAs (Additional file 1: Fig. S4). Among 51 patients who were followed up without treatment, there were no significant associations between disease progression and n-3 PUFAs, n-6 PUFAs, n-3/n-6 ratio, SFAs, or MUFAs (Additional file 1: Fig. S5).

## Discussion

In the present study, we found that patients with sarcoidosis had lower levels of n-3 PUFAs, n-6 PUFAs, and n-3/n-6 ratio than healthy controls. Furthermore, among the patients with sarcoidosis, lower levels of SFAs and n-3/n-6 ratio were associated with multiple organ involvement. Our data indicated that distinctive LCFA profiles were associated with sarcoidosis.

It is well-known that n-3 PUFAs have anti-inflammatory effects via several immune cells. For example, n-3 PUFAs prevented differentiation and activation of CD4^+^ T cells, a key component of sarcoidosis [10]. Incorporation of n-3 PUFAs induced changes in membrane domain organization, which could account for the alterations in CD4^+^ T cell function [21, 22]. In addition, n-3 PUFAs were reported to blunt M1 macrophage polarization and promote M2 polarization by decreasing the secretion of Th1 cytokines [10]. Meanwhile, n-3 PUFAs attenuate the differentiation and activation of Th17 cells, which are recognized to play important roles in the pathogenesis of sarcoidosis. Several studies demonstrated that addition of n-3 PUFAs resulted in a decrease in Th17 differentiation from CD4^+^ T cells [23, 24]. Possible mechanisms include failure of CD4^+^ T cells to activate Stat-3 in response to pro-TH17 signals under high levels of n-3 PUFAs [25, 26]. Meanwhile, supplementation of n-3 PUFAs promoted the accumulation and proliferation of regulatory T cells (Tregs) in mouse models [27, 28, 29]. Furthermore, n-3 PUFAs were shown to increase M2 macrophages, thereby inducing Treg differentiation [27, 30]. Given the anti-inflammatory roles of n-3 PUFAs, it is possible that the decreased levels of n-3 PUFAs promote immune cell activation, thus facilitating the development of sarcoidosis.

Meanwhile, the association of sarcoidosis with n-6 PUFAs is not easy to explain. This is because that n-6 PUFAs can play pro-inflammatory or anti-inflammatory roles, depending on the types of lipid mediators, receptors, and target immune cells [11]. For example, PGE_2_, a representative lipid mediator derived from n-6 PUFAs, promoted inflammation via EP2 receptors on neutrophils and tumor-associated fibroblasts [31]. However, PGE_2_ signaling via EP4 receptors had anti-inflammatory roles by regulating Th1 cytokine production in a colitis model [32]. Similarly, leukotriene B4 (LTB_4_), another important lipid mediator derived from n-6 PUFAs, promoted the production of inflammatory cytokines by stimulating BTL1 receptors on dendritic cells, but suppressed the production of inflammatory cytokines by stimulating BLT2 receptors on cryptic cells in colitis models [33, 34]. Although the precise roles of n-6 PUFAs for sarcoidosis are unknown, it is possible that the decreased levels of n-6 PUFAs indicate attenuated anti-inflammatory properties.

When considering immunomodulatory roles of LCFAs, the balance between n-3 and n-6 PUFAs is also important. The anti-inflammatory properties of n-3 PUFAs are accomplished by competing against n-6 PUFAs. Lipid mediators derived from n-3 and n-6 PUFAs are orchestrated by cyclooxygenase, lipoxygenase, or cytochrome p450 enzymes [35]. In the presence of n-3 PUFAs, the competition for the enzymes reduces the synthesis of n-6 PUFAs. Therefore, the balance between n-3 and n-6 PUFAs, expressed as the n-3/n-6 ratio, is considered to be an index of inflammation. The lower level of n-3/n-6 ratio in the patients with sarcoidosis and the multiple organ involvement in the present study may indicate an increased pro-inflammatory status in these patients.

The potential mechanisms for the effects of SFAs on immune systems were reported to include protection from pathogenic microorganisms possibly by activating the Toll-like receptor 4 signaling pathway [36]. Therefore, lower levels of SFAs were associated with poor outcomes in infectious diseases, while supplementation of SFAs improved these outcomes [37, 38, 39, 40]. However, the underlying mechanisms for the association of decreased levels of SFAs with multiple organ involvement in patients with sarcoidosis remain unknown.

There are three major limitations to the present study. First, there was a potential selection bias because we included patients with sarcoidosis who had available fasting serum samples at diagnosis. The blood samples were collected when the patients were admitted to our hospital for diagnosis and evaluation of sarcoidosis. Therefore, asymptomatic and/or mild cases who did not require admission could have been missed in the study. Second, it is unknown whether the differences in LCFA levels were associated with the development and/or disease activity of sarcoidosis, or merely resulted from deterioration of the nutritional status due to sarcoidosis. Further prospective studies that evaluate the time-course changes in LCFAs during disease progression or improvement are warranted to clarify the associations between LCFAs and disease activity in sarcoidosis. Third, the differences in biological activities among the individual LCFAs are unknown. Usually, single LCFAs in the same structural group are considered to have similar biological activities; however, the precise differences among those LCFAs have not been fully investigated (for example, among linolenic acid, eicosapentaenoic acid, docosapentaenoic acid, and docosahexaenoic acid in the n-3 PUFAs). In fact, LCFAs in the same structural group were strongly associated with one another, and therefore had similar associations with sarcoidosis and/or multiple organ involvement. However, at the same time, some LCFAs were weakly associated with other LCFAs in the same structural group, and demonstrated different associations with sarcoidosis and/or multiple organ involvement. Further studies are needed to elucidate the mechanisms and clinical significance of LCFAs for sarcoidosis.

## Conclusions

Lower levels of n-3 PUFAs, n-6 PUFAs, and n-3/n-6 ratio were associated with sarcoidosis, and lower levels of SFAs and n-3/n-6 ratio were associated with multiple organ involvement. Assessment of LCFA profiles may be useful for the diagnosis of sarcoidosis and evaluation of the disease activity.

## Supplementary Information

**Additional file 1**. **Supplementary Figure 1**: Correlation between the levels of long-chain fatty acids and clinical data. **Supplementary Figure 2:** Correlation between the levels of each long-chain fatty acid and clinical data. **Supplementary Figure 3:** Comparison of the levels of each long-chain fatty acid between healthy subjects and sarcoidosis patients. **Supplementary Figure 4:** Associations of the levels of long-chain fatty acids with the radiographic stage. **Supplementary Figure 5:** Associations of the levels of long-chain fatty acids with clinical course in 51 patients without treatments. **Supplementary Table 1.** Receiver operating characteristic curve analysis for the levels of long-chain fatty acids to predict sarcoidosis. **Supplementary Table 2.** Receiver operating characteristic curve analysis for the levels of long-chain fatty acids to predict affected multiple organs of sarcoidosis. **Supplementary Table 3.** Healthy controls and patients’ characteristics. **Supplementary Table 4.** Difference in lipid level between the sexes. **Supplementary Table 5.** Univariate logistic regression analyses of each long-chain fatty acid for the diagnosis of sarcoidosis. **Supplementary Table 6.** Univariate logistic regression analyses of each long-chain fatty acid for multiple organ involvements in sarcoidosis.

## Data Availability

The datasets used and/or analyzed during the current study are available from the corresponding author on reasonable request.

## References

[1] Statement on sarcoidosis (1999). Joint Statement of the American Thoracic Society (ATS), the European Respiratory Society (ERS) and the World Association of Sarcoidosis and Other Granulomatous Disorders (WASOG) adopted by the ATS Board of Directors and by the ER. Am J Respir Crit Care Med.

[2] Thillai M, Atkins CP, Crawshaw A, Hart SP, Ho LP, Kouranos V (2021). BTS clinical statement on pulmonary sarcoidosis. Thorax.

[3] Zhang H, Costabel U, Dai H (2021). The Role of Diverse Immune Cells in Sarcoidosis. Front Immunol.

[4] Erika L, Pearce, Edward J (2013). Pearce. Metabolic pathways in immune cell activation and quiescence. Immunity.

[5] Fox CJ, Hammerman PS, Thompson CB (2005). Fuel feeds function: Energy metabolism and the T-cell response. Nat Rev Immunol.

[6] Kishton RJ, Sukumar M, Restifo NP (2017). Metabolic regulation of T cell longevity and function in tumor immunotherapy. Cell Metab.

[7] O’Brien KL, Finlay DK (2019). Immunometabolism and natural killer cell responses. Nat Rev Immunol.

[8] Newton R, Priyadharshini B, Turka LA (2016). Immunometabolism of regulatory T cells. Nat Immunol.

[9] Lochner M, Berod L, Sparwasser T (2015). Fatty acid metabolism in the regulation of T cell function. Trends Immunol.

[10] Gutiérrez S, Svahn SL, Johansson ME. Effects of omega-3 fatty acids on immune cells. Int J Mol Sci. 2019;20. 10.3390/ijms20205028 PMC6834330 31614433

[11] Nagatake T, Kunisawa J (2019). Emerging roles of metabolites of ω3 and ω6 essential fatty acids in the control of intestinal inflammation. Int Immunol.

[12] Haghikia A, Jörg S, Duscha A, Berg J, Manzel A, Waschbisch A (2015). Dietary fatty acids directly impact central nervous system autoimmunity via the small intestine. Immunity.

[13] Hammer A, Schliep A, Jörg S, Haghikia A, Gold R, Kleinewietfeld M (2017). Impact of combined sodium chloride and saturated long-chain fatty acid challenge on the differentiation of T helper cells in neuroinflammation. J Neuroinflammation.

[14] Weatherill AR, Lee JY, Zhao L, Lemay DG, Youn HS, Hwang DH (2005). Saturated and polyunsaturated fatty acids reciprocally modulate dendritic cell functions mediated through TLR4. J Immunol.

[15] van der Does AM, Heijink M, Mayboroda OA, Persson LJ, Aanerud M, Bakke P (2019). Dynamic differences in dietary polyunsaturated fatty acid metabolism in sputum of COPD patients and controls. Biochim Biophys Acta - Mol Cell Biol Lipids.

[16] Chu SG, Villalba JA, Liang X, Xiong K, Tsoyi K, Ith B (2019). Palmitic acid-rich high-fat diet exacerbates experimental pulmonary fibrosis by modulating endoplasmic reticulum stress. Am J Respir Cell Mol Biol.

[17] Scaioli E, Liverani E, Belluzzi A. The imbalance between N-6/N-3 polyunsaturated fatty acids and inflammatory bowel disease: A comprehensive review and future therapeutic perspectives. Int J Mol Sci. 2017;18. 10.3390/ijms18122619 PMC5751222 29206211

[18] Inoue Y, Inui N, Hashimoto D, Enomoto N, Fujisawa T, Nakamura Y, et al. Cumulative Incidence and Predictors of Progression in Corticosteroid-Naive Patients with Sarcoidosis. PLoS One. 2015;10. 10.1371/journal.pone.0143371 PMC4648534 26575272

[19] Hunninghake CW, Gilbert S, Pueringer R, Dayton C, Floerchinger C, Helmers R (1994). Outcome of the Treatment for Sarcoidosis. Am J Resp Crit Care.

[20] Huggins JT, Doelken P, Sahn SA, King L, Judson MA (2006). Pleural effusions in a series of 181 outpatients with sarcoidosis. Chest.

[21] Hou TY, Rola Barhoumid, Fan Y-Y, Rivera GM, Hannoush RN, McMurray DN (2016). n-3 polyunsaturated fatty acids suppress CD4 + T cell proliferation by altering phosphatidylinositol-(4,5)-bisphosphate [PI(4,5)P2] organization. Biochim Et Biophys Acta-Biomembr..

[22] Fan Y-Y, Natividad R, Fuentes, Hou TY, Barhoumi R, Li XC, Deutz NEP (2018). Remodelling of primary human CD4 + T cell plasma membrane order by n-3 PUFA. Br J Nutr.

[23] Chiurchiù V, Alessandro Leuti, Dalli J, Jacobsson A, Battistini L, Maccarrone M (2016). Pro-resolving lipid mediators Resolvin D1, Resolvin D2 and Maresin 1 are critical in modulating T cell responses. Sci Transl Med.

[24] Jeffery L, Fisk HL, Calder PC, Filer A, Raza K, Buckley CD (2017). Plasma levels of eicosapentaenoic acid are associated with anti-TNF responsiveness in rheumatoid arthritis and inhibit the etanercept-driven rise in Th17 cell differentiation in vitro. J Rheumatol.

[25] Monk JM, Hou TY, Turk HF, McMurray DN, Chapkin RS (2013). n3 PUFAs reduce mouse CD4 + T-Cell ex vivo polarization into Th17 Cells. J Nutr.

[26] Allen MJ, Fan YY, Monk JM, Hou TY, Barhoumi R, McMurray DN (2014). n-3 PUFAs reduce T-helper 17 cell differentiation by decreasing responsiveness to interleukin-6 in isolated mouse splenic CD4 + T cells. J Nutr.

[27] Onodera T, Fukuhara A, Shin J, Hayakawa T, Otsuki M, Shimomura I (2017). Eicosapentaenoic acid and 5-HEPE enhance macrophage-mediated Treg induction in mice. Sci Rep.

[28] Lian M, Luo W, Sui Y, Li Z, Hua J (2015). Dietary n-3 PUFA protects mice from Con a induced liver injury by modulating regulatory T cells and PPAR-γ expression. PLoS One.

[29] Woodworth HL, McCaskey SJ, Duriancik DM, Clinthorne JF, Langohr IM, Gardner EM (2010). Dietary fish oil alters T lymphocyte cell populations and exacerbates disease in a mouse model of inflammatory colitis. Cancer Res.

[30] Han SC, Koo DH, Kang NJ, Yoon WJ, Kang GJ, Kang HK (2015). Docosahexaenoic acid alleviates atopic dermatitis by generating tregs and IL-10/TGF-β-modified macrophages via a TGF-β-dependent mechanism. J Invest Dermatol.

[31] Aoki T, Narumiya S (2017). Prostaglandin E2-EP2 signaling as a node of chronic inflammation in the colon tumor microenvironment. Inflamm Regen.

[32] Kabashima K, Saji T, Murata T, Nagamachi M, Matsuoka T, Segi E (2002). The prostaglandin receptor EP4 suppresses colitis, mucosal damage and CD4 cell activation in the gut. J Clin Invest.

[33] Maj T, Wang W, Crespo J, Zhang H, Wang W, Zhao L (2018). Oxidative stress controls regulatory T cell apoptosis and suppressor activity and PD-L1-blockade resistance in tumor. Nat Immunol.

[34] Iizuka Y, Okuno T, Saeki K, Uozaki H, Okada S, Misaka T (2010). Protective role of the leukotriene B 4 receptor BLT2 in murine inflammatory colitis. FASEB J.

[35] Schwanke RC, Marcon R, Bento AF, Calixto JB (2016). EPA- and DHA-derived resolvins’ actions in inflammatory bowel disease. Eur J Pharmacol..

[36] Zhang RN, Pan Q, Zhang Z, Cao HX, Shen F, Fan JG. Saturated fatty acid inhibits viral replication in chronic hepatitis B virus infection with nonalcoholic fatty liver disease by toll-like receptor 4-mediated innate immune response. Hepat Mon. 2015;15. 10.5812/hepatmon.15(5)2015.27909 PMC4451278 26045709

[37] Wang L, Johnson EA (1992). InhibitionofListeriamonocytogenes byFattyAcids and Monoglycerides. Appl Env Microbiol.

[38] Kitahara T, Koyama N, Matsuda J, Aoyama Y, Hirakata Y, Kamihira S (2004). Antimicrobial activity of saturated fatty acids and fatty amines against methicillin-resistant Staphylococcus aureus. Biol Pharm Bull.

[39] Dingwoke EJ, Adamude FA, Chukwuocha CE, Ambi AA, Nwobodo NN, Sallau AB (2019). Inhibition of trypanosoma evansi protein-tyrosine phosphatase by myristic acid analogues isolated from khaya senegalensis and tamarindus indica. J Exp Pharmacol.

[40] Fischer CL, Drake DR, Dawson DV, Blanchette DR, Brogden KA, Wertz PW (2012). Antibacterial activity of sphingoid bases and fatty acids against gram-positive and gram-negative bacteria. Antimicrob Agents Chemother.

