# Supplementary material for: Associations of serum long-chain fatty acids with multiple organ involvement in patients with sarcoidosis
Source: BMC Pulm Med. 2022 Jul 28;22:290. doi: 10.1186/s12890-022-02084-x (PMC9335968; doi:10.1186/s12890-022-02084-x)
Supplement: Supplementary file 1 — Additional file 1. Supplementary Figure 1: Correlation between the levels of long-chain fatty acids and clinical data. Supplementary Figure 2: Correlation between the levels of each long-chain fatty acid and clinical data. Supplementary Figure 3: Comparison of the levels of each long-chain fatty acid between healthy subjects and sarcoidosis patients. Supplementary Figure 4: Associations of the levels of long-chain fatty acids with the radiographic stage. Supplementary Figure 5: Associations of the levels of long-chain fatty acids with clinical course in 51 patients without treatments. Supplementary Table 1. Receiver operating characteristic curve analysis for the levels of long-chain fatty acids to predict sarcoidosis. Supplementary Table 2. Receiver operating characteristic curve analysis for the levels of long-chain fatty acids to predict affected multiple organs of sarcoidosis. Supplementary Table 3. Healthy controls and patients’ characteristics. Supplementary Table 4. Difference in lipid level between the sexes. Supplementary Table 5. Univariate logistic regression analyses of each long-chain fatty acid for the diagnosis of sarcoidosis. Supplementary Table 6. Univariate logistic regression analyses of each long-chain fatty acid for multiple organ involvements in sarcoidosis. [file 12890_2022_2084_MOESM1_ESM.docx]

**Associations of serum long-chain fatty acids with multiple organ involvements in patients with sarcoidosis**

**Authors:** Takahito Suzuki, Masato Karayama, Yusuke Inoue, Hironao Hozumi, Yuzo Suzuki, Kazuki Furuhashi, Tomoyuki Fujisawa, Noriyuki Enomoto, Yutaro Nakamura, Naoki Inui, Takafumi Suda


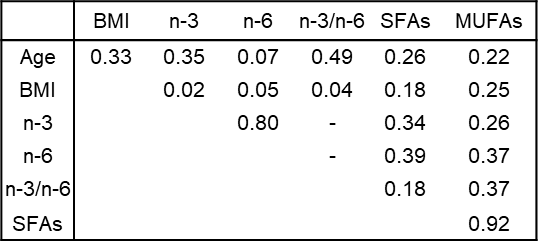
**Supplementary Figure 1: Correlation between the levels of long-chain fatty acids and clinical data.**

Data are expressed as Spearman’s rank correlation coefficient.

BMI, body mass index; n-3, n-3 polyunsaturated fatty acids; n-6, n-6 polyunsaturated fatty acids; n-3/n-6, n-3/n-6 ratio; SFAs, saturated fatty acids; MUFAs, monounsaturated fatty acids.

**
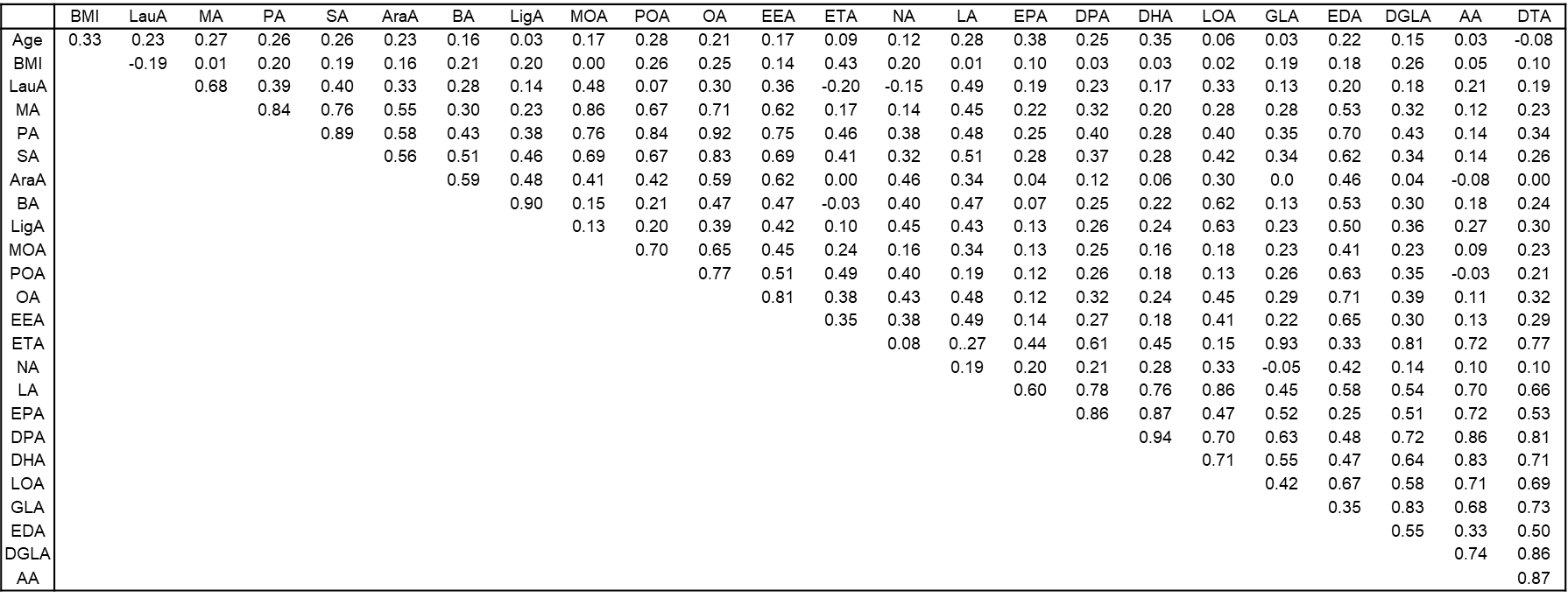
Supplementary Figure 2: Correlation between the levels of each long-chain fatty acid and clinical data.**

Data are expressed as Spearman’s rank correlation coefficient.

BMI, body mass index; LauA, lauric acid; MA, myristic acid; PA, palmitic acid; SA, stearic acid; AraA, arachidic acid; BA, behenic acid; LigA, lignoceric acid; MOA, myristoleic acid; POA, palmitoleic acid; OA, oleic acid; EEA, eicosenoic acid; ETA, eicosatrienoic acid; NA, nervonic acid; LA, linolenic acid; EPA, eicosapentanoic acid; DPA, docosapentaenoic acid; DHA, docosahexanoic acid; LOA, linoleic acid; GLA, γ-linolenic acid; EDA, eicosadienoic acid; DGLA, dihomo-γ-linolenic acid; AA, arachidonic acid; DTA, docosatetraenoic acid.

**Supplementary Figure 3: Comparison of the levels of each long-chain fatty acid between healthy subjects and sarcoidosis patients**


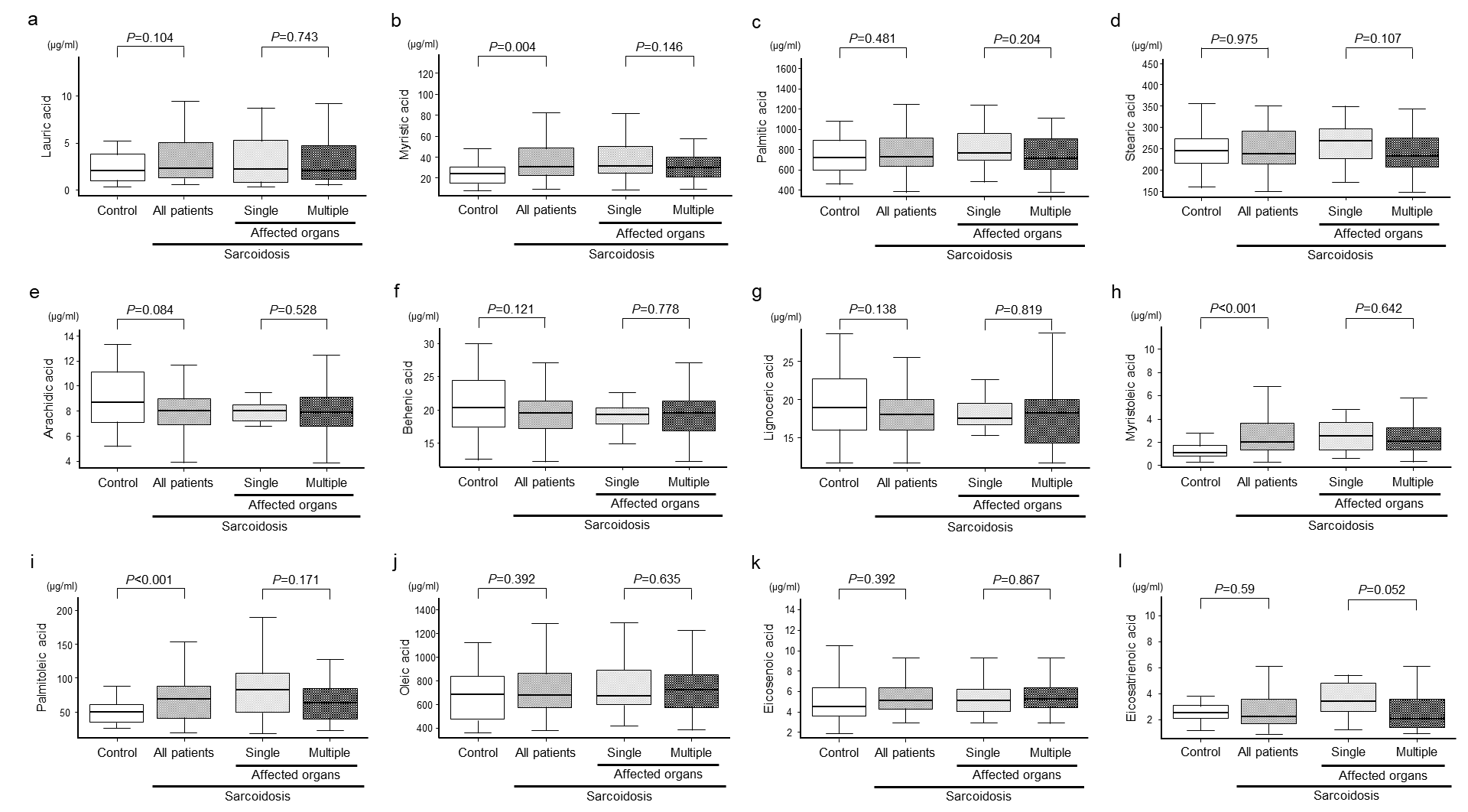


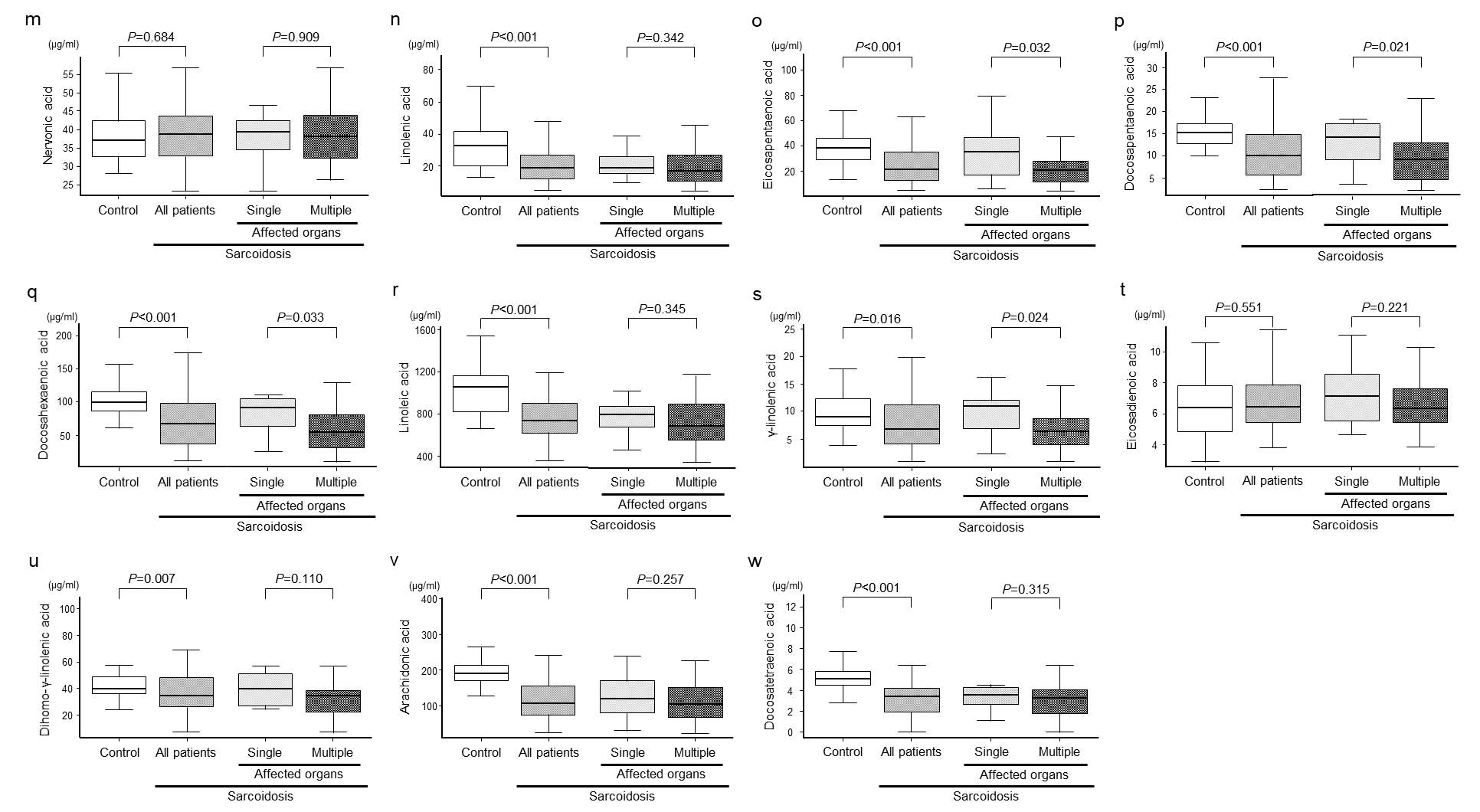


Horizontal lines, boxes, and error bars, represent the median, the 25^th^ and 75^th^ percentiles, and the 10^th^ and 90^th^ percentiles, respectively.

**
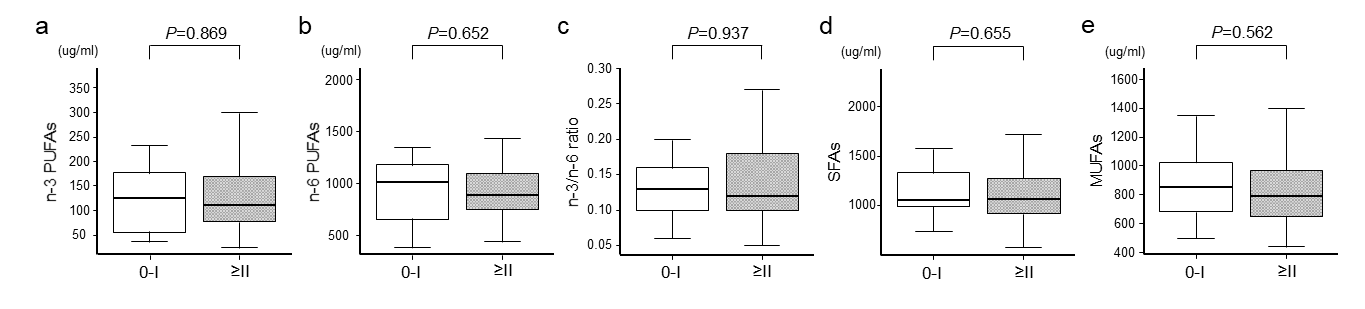
Supplementary Figure 4: Associations of the levels of long-chain fatty acids with the radiographic stage.**

Horizontal lines, boxes, and error bars, represent the median, the 25^th^ and 75^th^ percentiles, and the 10^th^ and 90^th^ percentiles, respectively.

Abbreviations: PUFAs, polyunsaturated fatty acids; SFAs, saturated fatty acids; MUFAs, monounsaturated fatty acids

**
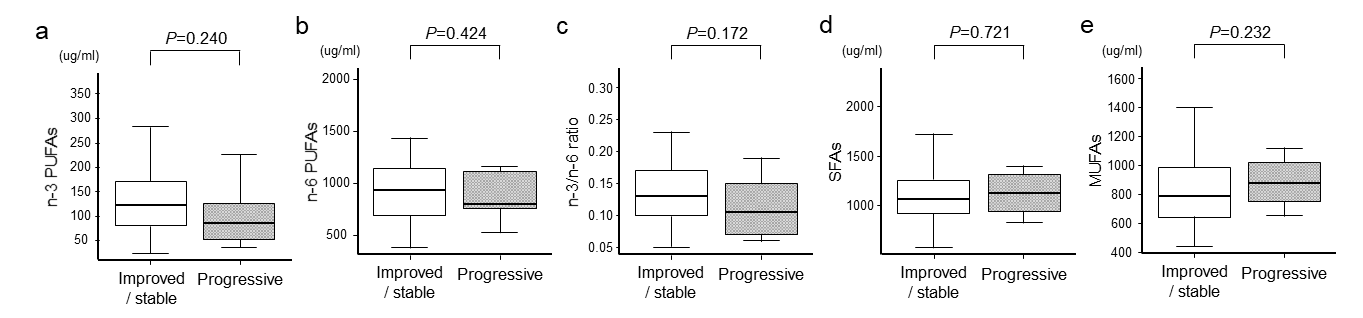
Supplementary Figure 5: Associations of the levels of long-chain fatty acids with clinical course in 51 patients without treatments.**

1. n-3 PUFAs, (b) n-6 PUFAs, (c) n-3/n-6 ratio (d) SFAs, (e) MUFAs

Horizontal lines, boxes, and error bars, represent the median, the 25^th^ and 75^th^ percentiles, and the 10^th^ and 90^th^ percentiles, respectively.

Abbreviations: PUFAs, polyunsaturated fatty acids; SFAs, saturated fatty acids; MUFAs, monounsaturated fatty acids.

**Supplementary** **Table 1. Receiver operating characteristic curve analysis for the levels of long-chain fatty acids to predict sarcoidosis**

|  | **Cut off value** | **AUC** | **95%CI** | **Sensitivity**  **(%)** | **Specificity**  **(%)** |
| --- | --- | --- | --- | --- | --- |
| n-3 PUFAs | 136.0 | 0.77 | 0.68-0.86 | 63.5 | 92.1 |
| n-6 PUFAs | 1236.0 | 0.82 | 0.74-0.90 | 92.1 | 57.9 |
| n-3/n-6 ratio | 0.11 | 0.59 | 0.48-0.70 | 39.7 | 81.6 |
| SFAs | 897.5 | 0.54 | 0.42-0.66 | 84.1 | 31.6 |
| MUFAs | 562.8 | 0.57 | 0.46-0.69 | 90.5 | 28.9 |
| Lauric acid | 1.6 | 0.60 | 0.48-0.71 | 71.4 | 47.4 |
| Myristic acid | 30.3 | 0.67 | 0.57-0.78 | 54.0 | 76.3 |
| Palmitic acid | 655.0 | 0.54 | 0.42-0.66 | 73.0 | 39.5 |
| Stearic acid | 230.5 | 0.50 | 0.38-0.62 | 44.4 | 68.4 |
| Arachidic acid | 9.5 | 0.60 | 0.48-0.72 | 84.1 | 44.7 |
| Behenic acid | 22.7 | 0.59 | 0.47-0.71 | 85.7 | 39.5 |
| Lignoceric acid | 18.3 | 0.59 | 0.47-0.71 | 60.3 | 57.9 |
| Myristoleic acid | 1.8 | 0.73 | 0.63-0.83 | 63.5 | 76.3 |
| Palmitoleic acid | 67.2 | 0.71 | 0.62-0.81 | 55.6 | 89.5 |
| Oleic acid | 805.4 | 0.45 | 0.33-0.57 | 69.8 | 34.2 |
| Eicosenoic acid | 4.6 | 0.59 | 0.47-0.71 | 71.4 | 52.6 |
| Eicosatrienoic acid | 2.0 | 0.54 | 0.40-0.67 | 44.7 | 76.3 |
| Nervonic acid | 43.1 | 0.52 | 0.41-0.64 | 28.6 | 84.2 |
| Linolenic acid | 22.2 | 0.74 | 0.65-0.84 | 66.7 | 71.1 |
| Eicosapentaenoic acid | 29.1 | 0.72 | 0.62-0.82 | 66.7 | 76.3 |
| Docosapentaenoic acid | 11.3 | 0.75 | 0.65-0.84 | 61.9 | 92.1 |
| Docosahexaenoic acid | 72.2 | 0.77 | 0.68-0.86 | 61.9 | 92.1 |
| Linoleic acid | 914.4 | 80.3 | 0.72-0.89 | 81.0 | 65.8 |
| γ-linolenic acid | 6.8 | 0.64 | 0.54-0.75 | 52.4 | 81.6 |
| Eicosadienoic acid | 5.1 | 0.54 | 0.42-0.66 | 85.7 | 28.9 |
| Dihomo-γ-linolenic acid | 35.4 | 0.66 | 0.56-0.77 | 58.7 | 78.9 |
| Arachidonic acid | 159.8 | 0.87 | 0.80-0.94 | 81.0 | 92.1 |
| Docosatetraenoic acid | 4.1 | 0.82 | 0.73-0.90 | 74.6 | 86.8 |

AUC, area under the curve; CI, Confidence Interval; PUFAs, polyunsaturated fatty acids; SFAs, saturated fatty acids; MUFAs, monounsaturated fatty acids.

**Supplementary** **Table 2. Receiver operating characteristic curve analysis for the levels of long-chain fatty acids to predict affected multiple organs of sarcoidosis**

|  | **Cut off value** | **AUC** | **95%CI** | **Sensitivity**  **(%)** | **Specificity**  **(%)** |
| --- | --- | --- | --- | --- | --- |
| n-3 PUFAs | 136.0 | 0.67 | 0.52-0.82 | 73.3 | 61.1 |
| n-6 PUFAs | 843.2 | 0.60 | 0.45-0.74 | 48.9 | 77.8 |
| n-3/n-6 ratio | 0.14 | 0.68 | 0.52-0.84 | 75.6 | 66.7 |
| SFAs | 991.7 | 0.61 | 0.46-0.77 | 44.4 | 88.9 |
| MUFAs | 562.8 | 0.46 | 0.29-0.62 | 91.1 | 11.1 |
| Lauric acid | 4.5 | 0.47 | 0.30-0.64 | 71.1 | 38.9 |
| Myristic acid | 22.2 | 0.55 | 0.39-0.71 | 28.9 | 88.9 |
| Palmitic acid | 689.1 | 0.60 | 0.45-0.76 | 44.4 | 88.9 |
| Stearic acid | 282.9 | 0.63 | 0.48-0.79 | 77.8 | 50.0 |
| Arachidic acid | 6.8 | 0.55 | 0.41-0.70 | 31.1 | 94.4 |
| Behenic acid | 20.5 | 0.48 | 0.33-0.63 | 35.6 | 77.8 |
| Lignoceric acid | 17.7 | 0.52 | 0.37-0.67 | 62.2 | 61.1 |
| Myristoleic acid | 2.9 | 0.54 | 0.37-0.71 | 73.3 | 50.0 |
| Palmitoleic acid | 87.0 | 0.61 | 0.45-0.78 | 82.2 | 44.4 |
| Oleic acid | 678.0 | 0.46 | 0.30-0.62 | 53.3 | 55.6 |
| Eicosenoic acid | 6.3 | 0.51 | 0.35-0.68 | 33.3 | 77.8 |
| Eicosatrienoic acid | 2.5 | 0.71 | 0.52-0.90 | 67.9 | 80.0 |
| Nervonic acid | 33.5 | 0.49 | 0.34-0.64 | 33.3 | 83.3 |
| Linolenic acid | 15.1 | 0.58 | 0.43-0.73 | 46.7 | 77.8 |
| Eicosapentaenoic acid | 32.4 | 0.68 | 0.52-0.83 | 82.2 | 61.1 |
| Docosapentaenoic acid | 11.2 | 0.69 | 0.54-0.83 | 73.3 | 72.2 |
| Docosahexaenoic acid | 71.5 | 0.67 | 0.53-0.82 | 71.1 | 66.7 |
| Linoleic acid | 672.8 | 0.58 | 0.43-0.72 | 46.7 | 83.3 |
| γ-linolenic acid | 6.8 | 0.68 | 0.54-0.83 | 64.4 | 77.8 |
| Eicosadienoic acid | 6.5 | 0.60 | 0.44-0.76 | 62.2 | 61.1 |
| Dihomo-γ-linolenic acid | 39.2 | 0.63 | 0.48-0.78 | 77.8 | 55.6 |
| Arachidonic acid | 55.6 | 0.59 | 0.43-0.75 | 24.4 | 94.4 |
| Docosatetraenoic acid | 1.8 | 0.58 | 0.43-0.73 | 31.1 | 94.4 |

AUC, area under the curve; CI, Confidence Interval; PUFAs, polyunsaturated fatty acids; SFAs, saturated fatty acids; MUFAs, monounsaturated fatty acids.

**Supplementary Table 3. Healthy controls and patients’ characteristics**

|  | **Healthy controls**  **n=38** | **Patients with sarcoidosis**  **n=63** | ***P*-value** |
| --- | --- | --- | --- |
| Age, years | 34 (27-43) | 58 (45-68) | <0.001 |
| Sex, female | 15 (39.5) | 38 (60.3) | 0.064 |
| Body mass index, kg/m^2^ | 22.2 (19.6-23.7) | 21.4 (20.1-23.8) | 0.682 |
| Smoking, ever-smoker | 0 (0.0) | 31 (49.2) | <0.001 |

Data are presented as median (interquartile range) or number (%).

**Supplementary Table 4. Difference in lipid level between the sexes**

|  | **All**  **n=63** | **Males**  **n=25** | **Females**  **n=38** | ***p*-value** |
| --- | --- | --- | --- | --- |
| n-3 PUFAs | 122.9 (76.4-170.9) | 111.0 (76.0-195.8) | 123.1 (80.7-159.1) | 0.754 |
| n-6 PUFAs | 906.0 (719.0-1123.8) | 935.5 (797.0-1144.3) | 865.0 (639.8-1098.4) | 0.205 |
| n-3/n-6 ratio | 0.12 (0.10-0.17) | 0.11 (0.08-0.19) | 0.13 (0.11-0.17) | 0.414 |
| SFAs | 1060.5 (930.8-1287.0) | 1062.3 (941.3-1317.5) | 1022.9 (929.6-1245.1) | 0.763 |
| MUFAs | 802.4 (665.3-995.1) | 832.6 (651.5-1023.0) | 792.1 (672.8-968.8) | 0.712 |
| Lauric acid | 2.4 (1.4-5.1) | 2.3 (1.3-4.5) | 2.8 (1.7-5.5) | 0.431 |
| Myristic acid | 30.8 (22.4-48.7) | 30.8 (22.4-50.2) | 30.6 (22.6-39.4) | 0.938 |
| Palmitic acid | 724.1 (636.2-818.8) | 724.1 (655.0-925.5) | 723.1 (731.4-868.1) | 0.850 |
| Stearic acid | 238.4 (214.3-291.4) | 256.0 (215.8-291.2) | 235.8 (213.8-289.4) | 0.741 |
| Arachidic acid | 8.0 (6.9-9.0) | 8.0 (6.9-8.5) | 8.0 (6.9-9.3) | 0.658 |
| Behenic acid | 19.5 (17.2-21.4) | 19.2 (18.6-21.1) | 19.7 (15.9-22.0) | 0.668 |
| Lignoceric acid | 18.0 (16.0-20.0) | 18.2 (16.6-20.4) | 17.7 (14.5-19.5) | 0.209 |
| Myristoleic acid | 2.0 (1.3-3.6) | 2.3 (1.2-3.7) | 2.0 (1.5-3.4) | 0.950 |
| Palmitoleic acid | 69.8 (41.6-89.0) | 69.8 (40.8-85.5) | 73.7 (44.8-96.0) | 0.496 |
| Oleic acid | 678.0 (575.7-860.8) | 729.5 (565.4-890.7) | 674.4 (580.1-790.0) | 0.601 |
| Eicosenoic acid | 5.1 (4.3-6.4) | 5.8 (4.1-6.9) | 5.1 (4.4-6.1) | 0.452 |
| Eicosatrienoic acid | 2.3 (1.7-3.6) | 2.7 (2.0-3.6) | 2.0 (1.5-3.6) | 0.314 |
| Nervonic acid | 38.6 (32.8-43.7) | 39.3 (33.5-43.1) | 37.7 (32.5-43.7) | 0.694 |
| Linolenic acid | 18.9 (12.2-27.0) | 18.1 (11.0-29.7) | 19.1 (12.3-25.7) | 0.839 |
| Eicosapentaenoic acid | 21.6 (12.6-35.1) | 22.5 (12.6-49.4) | 21.2 (13.0-33.7) | 0.790 |
| Docosapentaenoic acid | 10.0 (5.7-14.8) | 11.2 (5.7-16.6) | 9.3 (5.7-14.2) | 0.346 |
| Docosahexaenoic acid | 66.6 (37.4-98.0) | 65.0 (30.5-101.7) | 68.0 (41.5-93.1) | 0.840 |
| Linoleic acid | 721.1 (609.9-890.4) | 796.2 (672.8-908.4) | 685.8 (546.8-871.7) | 0.232 |
| γ-linolenic acid | 6.8 (4.2-11.2) | 7.8 (5.2-11.2) | 6.5 (4.1-11.0) | 0.160 |
| Eicosadienoic acid | 6.4(5.5-7.9) | 6.5 (5.5-7.9) | 6.4 (5.3-8.1) | 0.916 |
| Dihomo-γ-linolenic acid | 34.4 (26.2-48.1) | 35.1 (30.9-49.6) | 32.0 (23.0-40.3) | 0.135 |
| Arachidonic acid | 106.2 (74.8-155.7) | 135.1 (85.5-184.4) | 100.0 (60.1-148.3) | 0.085 |
| Docosatetraenoic acid | 3.4 (1.9-4.2) | 3.6 (2.9-4.1) | 2.9 (1.7-4.3) | 0.102 |

Data are presented as median (interquartile range). Data are expressed in ug/mL except n-3/n-6 ratio.

PUFAs, polyunsaturated fatty acids; SFAs, saturated fatty acids; MUFAs, monounsaturated fatty acids.

**Supplementary** **table 5. Univariate logistic regression analyses of each long-chain fatty acid for the diagnosis of sarcoidosis**

|  | **Unadjusted** | | **Adjusted*** | |
| --- | --- | --- | --- | --- |
| **Variables, high** | **OR (95%CI)** | ***P*-value** | **OR (95%CI)** | ***P*-value** |
| Lauric acid | 2.08 (0.91-4.80) | 0.084 | 1.67 (0.60-4.68) | 0.331 |
| Myristic acid | 3.54 (1.45-8.69) | 0.006 | 2.50 (0.85-7.38) | 0.098 |
| Palmitic acid | 1.63 (0.70-3.81) | 0.259 | 0.76 (0.25-2.31) | 0.626 |
| Stearic acid | 0.58 (0.25-1.34) | 0.202 | 0.19 (0.05-0.72) | 0.014 |
| Arachidic acid | 0.23 (0.09-0.59) | 0.002 | 0.09 (0.02-0.38) | <0.001 |
| Behenic acid | 0.26 (0.10-0.67) | 0.005 | 0.12 (0.03-0.45) | 0.002 |
| Lignoceric acid | 0.48 (0.21-1.08) | 0.077 | 0.14 (0.04-0.52) | 0.003 |
| Myristoleic acid | 4.90 (1.99-12.10) | <0.001 | 5.98 (1.88-19.00) | 0.002 |
| Palmitoleic acid | 9.97 (3.16-31.40) | <0.001 | 6.03 (1.66-21.90) | 0.006 |
| Oleic acid | 0.83 (0.35-1.96) | 0.672 | 0.70 (0.23-2.15) | 0.527 |
| Eicosenoic acid | 2.57 (1.12-5.92) | 0.026 | 1.47 (0.49-4.40) | 0.489 |
| Eicosatrienoic acid | 0.38 (0.14-1.03) | 0.056 | 0.29 (0.07-1.23) | 0.093 |
| Nervonic acid | 1.97 (0.70-5.54) | 0.199 | 1.10 (0.31-3.93) | 0.886 |
| Linolenic acid | 0.20 (0.08-0.49) | <0.001 | 0.02 (0.00-0.13) | <0.001 |
| Eicosapentaenoic acid | 0.16 (0.06-0.39) | <0.001 | 0.03 (0.01-0.16) | <0.001 |
| Docosapentaenoic acid | 0.05 (0.01-0.19) | <0.001 | 0.01 (0.00-0.05) | <0.001 |
| Docosahexaenoic acid | 0.05 (0.01-0.19) | <0.001 | 0.01 (0.00-0.08) | <0.001 |
| Linoleic acid | 0.12 (0.05-0.31) | <0.001 | 0.06 (0.02-0.24) | <0.001 |
| γ-linolenic acid | 0.21 (0.08-0.54) | 0.001 | 0.11 (0.03-0.41) | 0.001 |
| Eicosadienoic acid | 2.16 (0.82-5.72) | 0.121 | 1.24 (0.29-5.21) | 0.772 |
| Dihomo-γ-linolenic acid | 0.19 (0.07-0.47) | <0.001 | 0.07 (0.02-0.27) | <0.001 |
| Arachidonic acid | 0.02 (0.01-0.08) | <0.001 | 0.01 (0.00-0.08) | <0.001 |
| Docosatetraenoic acid | 0.05 (0.02-0.16) | <0.001 | 0.06 (0.02-0.22) | <0.001 |

The cutoff value for each long-chain fatty acid is determined by Youden Index in receiver operating characteristic analysis (Supplementary Table 1). OR, odds ratio; CI, Confidence Interval.

*The odds ratio adjusted by age and sex.

**Supplementary table 6. Univariate logistic regression analyses of each long-chain fatty acid for multiple organ involvements in sarcoidosis**

|  | **Unadjusted** | | **Adjusted*** | |
| --- | --- | --- | --- | --- |
| **Variables, high** | **OR (95%CI)** | ***P*-value** | **OR (95%CI)** | ***P*-value** |
| Lauric acid | 0.64 (0.20-2.01) | 0.443 | 0.79 (0.22-2.78) | 0.708 |
| Myristic acid | 0.31 (0.06-1.53) | 0.150 | 0.56 (0.10-3.21) | 0.511 |
| Palmitic acid | 0.16 (0.03-0.76) | 0.022 | 0.20 (0.04-1.03) | 0.054 |
| Stearic acid | 0.29 (0.09-0.91) | 0.034 | 0.37 (0.11-1.22) | 0.101 |
| Arachidic acid | 0.13 (0.02-1.08) | 0.059 | 0.14 (0.02-1.28) | 0.082 |
| Behenic acid | 1.75 (0.49-6.25) | 0.389 | 2.12 (0.54-8.29) | 0.281 |
| Lignoceric acid | 2.36 (0.77-7.22) | 0.133 | 3.41 (0.97-11.90) | 0.055 |
| Myristoleic acid | 0.36 (0.12-1.13) | 0.081 | 0.41 (0.13-1.35) | 0.142 |
| Palmitoleic acid | 0.27 (0.08-0.90) | 0.033 | 0.19 (0.05-0.78) | 0.021 |
| Oleic acid | 1.31 (0.44-3.92) | 0.633 | 1.39 (0.42-4.61) | 0.587 |
| Eicosenoic acid | 1.27 (0.35-4.64) | 0.715 | 1.74 (0.42-7.13) | 0.444 |
| Eicosatrienoic acid | 0.12 (0.02-0.68) | 0.016 | 0.12 (0.02-0.82) | 0.030 |
| Nervonic acid | 0.40 (0.10-1.60) | 0.195 | 0.31 (0.07-1.43) | 0.132 |
| Linolenic acid | 0.33 (0.09-1.15) | 0.081 | 0.35 (0.09-1.30) | 0.116 |
| Eicosapentaenoic acid | 0.14 (0.04-0.47) | 0.001 | 0.16 (0.04-0.62) | 0.008 |
| Docosapentaenoic acid | 0.14 (0.04-0.48) | 0.002 | 0.16 (0.04-0.63) | 0.009 |
| Docosahexaenoic acid | 0.20 (0.06-0.66) | 0.008 | 0.27 (0.08-0.99) | 0.047 |
| Linoleic acid | 0.23 (0.06-0.90) | 0.035 | 0.29 (0.07-1.23) | 0.093 |
| γ-linolenic acid | 0.16 (0.04-0.56) | 0.004 | 0.19 (0.05-0.70) | 0.013 |
| Eicosadienoic acid | 0.39 (0.13-1.19) | 0.097 | 0.42 (0.13-1.39) | 0.157 |
| Dihomo-γ-linolenic acid | 0.23 (0.07-0.73) | 0.013 | 0.25 (0.07-0.84) | 0.025 |
| Arachidonic acid | 0.18 (0.02-1.53) | 0.116 | 0.21 (0.02-1.97) | 0.173 |
| Docosatetraenoic acid | 0.13 (0.02-1.08) | 0.059 | 0.15 (0.02-1.32) | 0.088 |

The cutoff value for each long-chain fatty acid is determined by Youden Index in receiver operating characteristic analysis (Supplementary Table 2). OR, odds ratio; CI, Confidence Interval.

*The odds ratio adjusted age, sex and serum angiotensin-converting enzyme.
